# Supplementary material for: Shiftless Is a Novel Member of the Ribosome Stress Surveillance Machinery That Has Evolved to Play a Role in Innate Immunity and Cancer Surveillance
Source: Viruses. 2023 Nov 23;15(12):2296. doi: 10.3390/v15122296 (PMC10747187; doi:10.3390/v15122296)
Supplement: Supplementary file 1 [file viruses-15-02296-s001.zip › viruses-2732250-supplementary.pdf]

Supplemental Materials

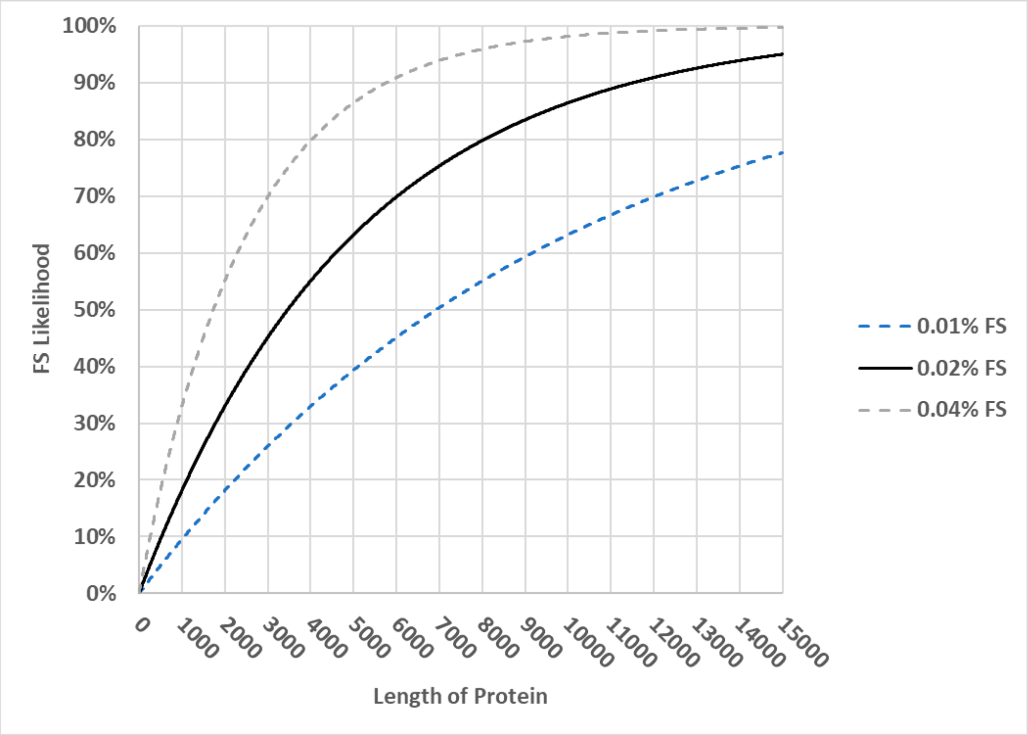

Supplemental Figure S1. Probability plot of the likelihood of a spontaneous ribosomal frameshift as a function of codon length. The plot was calculated with Excel using the following equation: P= 1-((1-rate of spontaneous frameshifting)^amino acid length).

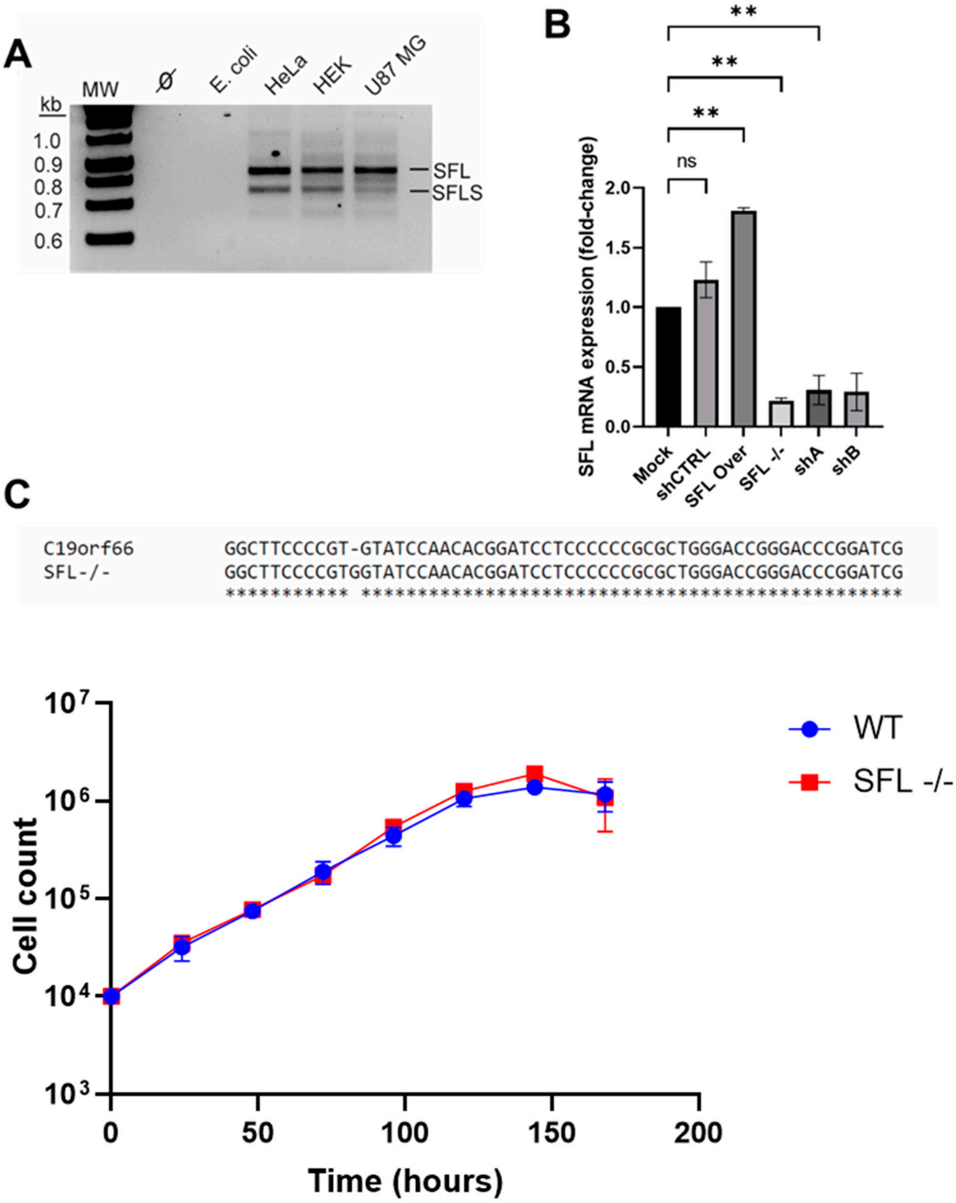

Supplemental Figure S2. Modifying SFL expression in the cell.

A. RT-PCR analysis of SFL in a blank sample (Ø), and RNA extracted from *E. coli*, HeLa, HEK293T and U87 MG cells. SFL denotes the full-length Shiftless mRNA while SFLS denotes a shorter Shiftless splice isoform. **B.** qRT-PCR analysis of SFL expression in HEK293T cells. **C.** Sequence validation of SFL$^{-/-}$ HEK293T cells. Yellow box indicates insertion of a G residue into the SFL coding sequence by CRISPR. **D.** SFL knockout does not alter cell growth. Growth curve of WT HEK293T cells (blue) and SFL-/- HEK293T cells (red).

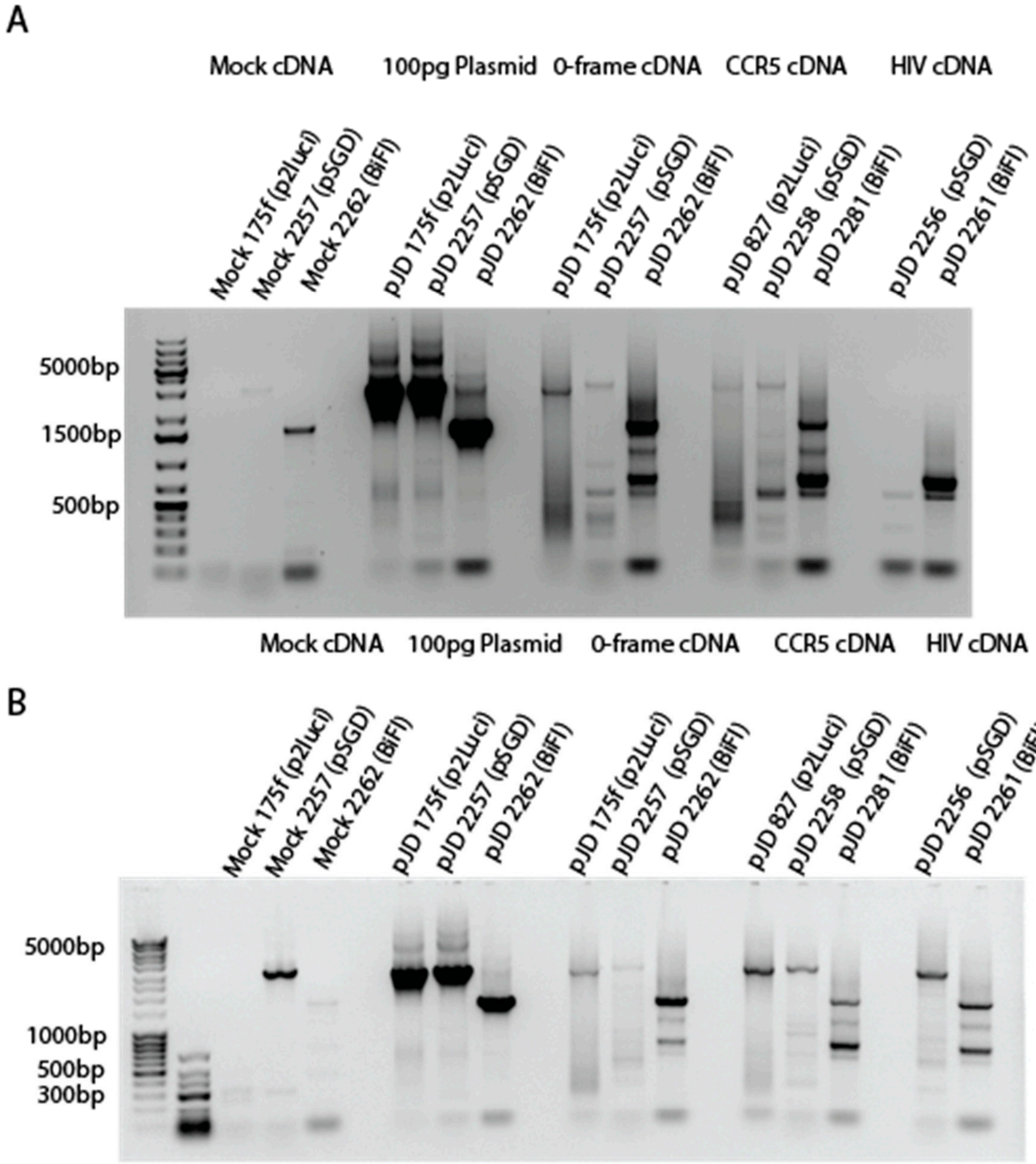

Supplemental Figure S3. Assessment of reporter splicing. RT-PCR analysis of plasmid DNA controls or mRNA extracted from HEK293T cells expressing first-generation dual luciferase (p2Luci), second-generation dual luciferase (pSGD), or bifluorescent (BiFl) reporters containing a 0-frame control, CCR5 -1 frameshift element, or HIV-1 -1 frameshift element and analyzed by gel electrophoresis. A and B denote two independent replicates.

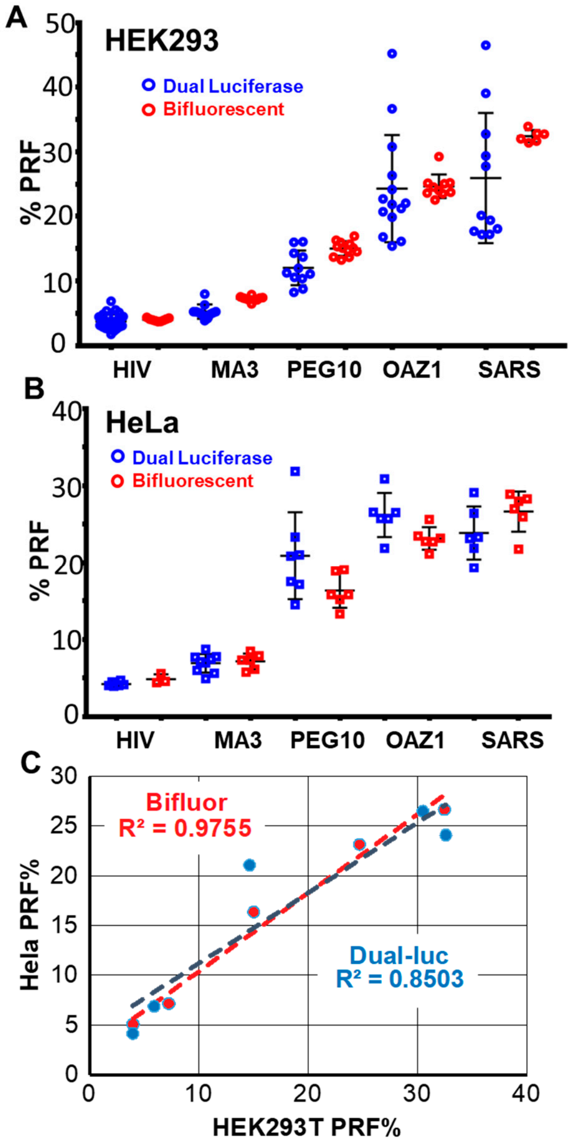

Supplemental Figure S4. Comparison of translational recoding measured with dual luciferase and bifluorescent reporters.

A, B: -1 PRF (HIV, MA3, PEG10, SARS) and +1 PRF (OAZ1 were measured in HEK93 or HeLa cells as indicated. Each dot indicates three technical replicates of one independent biological replicate. Bars indicate standard error. **C:** Linear regression analyses of the data compared between the two assay systems.

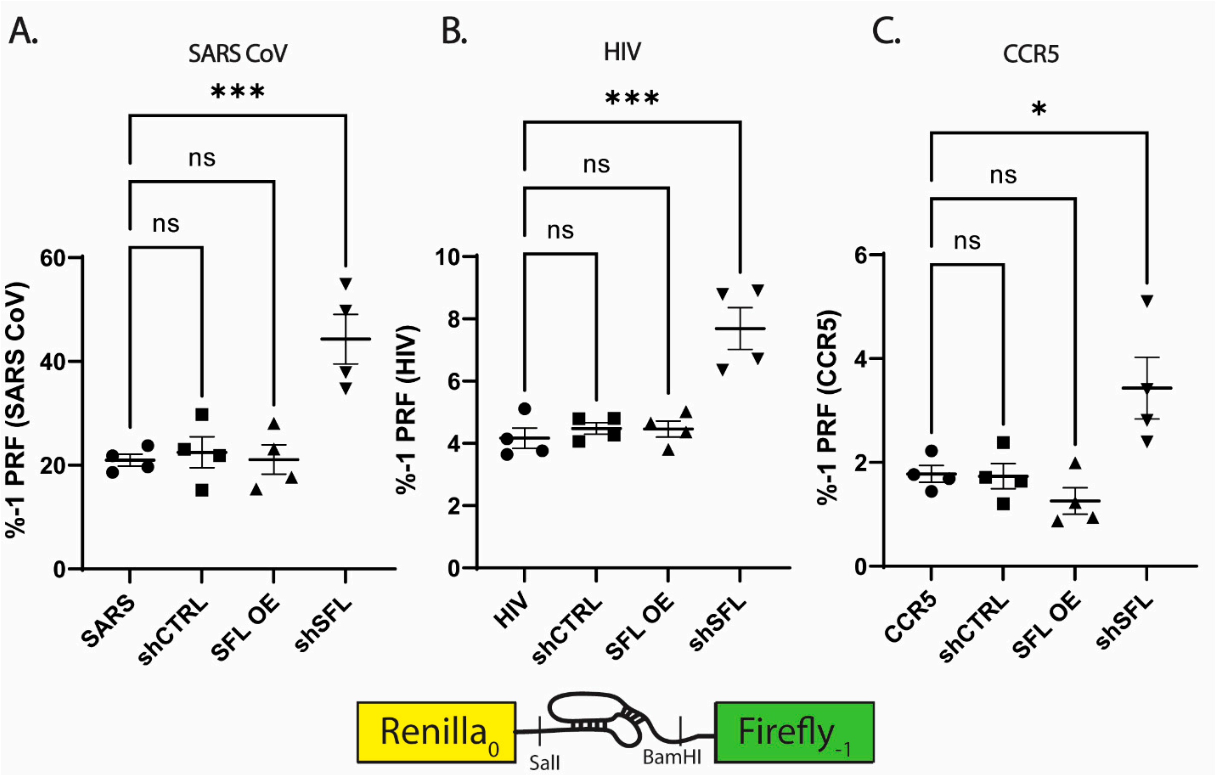

Supplemental Figure S5. SFL overexpression or knockdown alters -1 PRF.

Frameshift efficiency of three translational recoding elements measured using dual luciferase reporters in HEK293T cells over or under-expressing SFL. (A) SARS-CoV -1 PRF signal, (B) HIV-1 -1 PRF signal, (C) CCR5 -1 PRF signal.

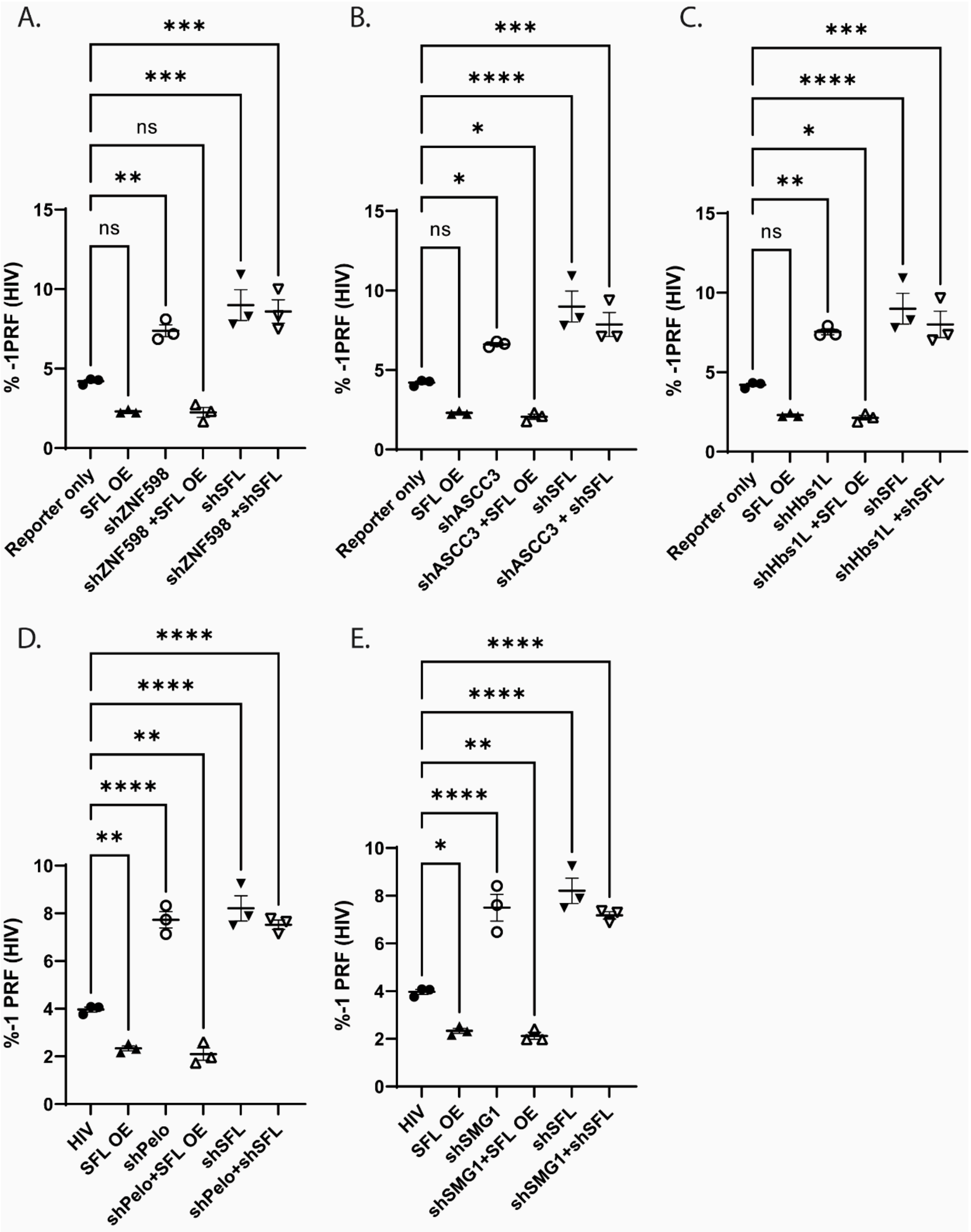

Supplemental Figure S6.

Frameshift efficiency of the HIV-1 -1 PRF signal in HEK293T cells over or under-expressing SFL in combination with shRNA knockdown of (A) ZNF598, (B) ASCC3, (C) Hbs1L, (D) Pelota, (E) SMG1.

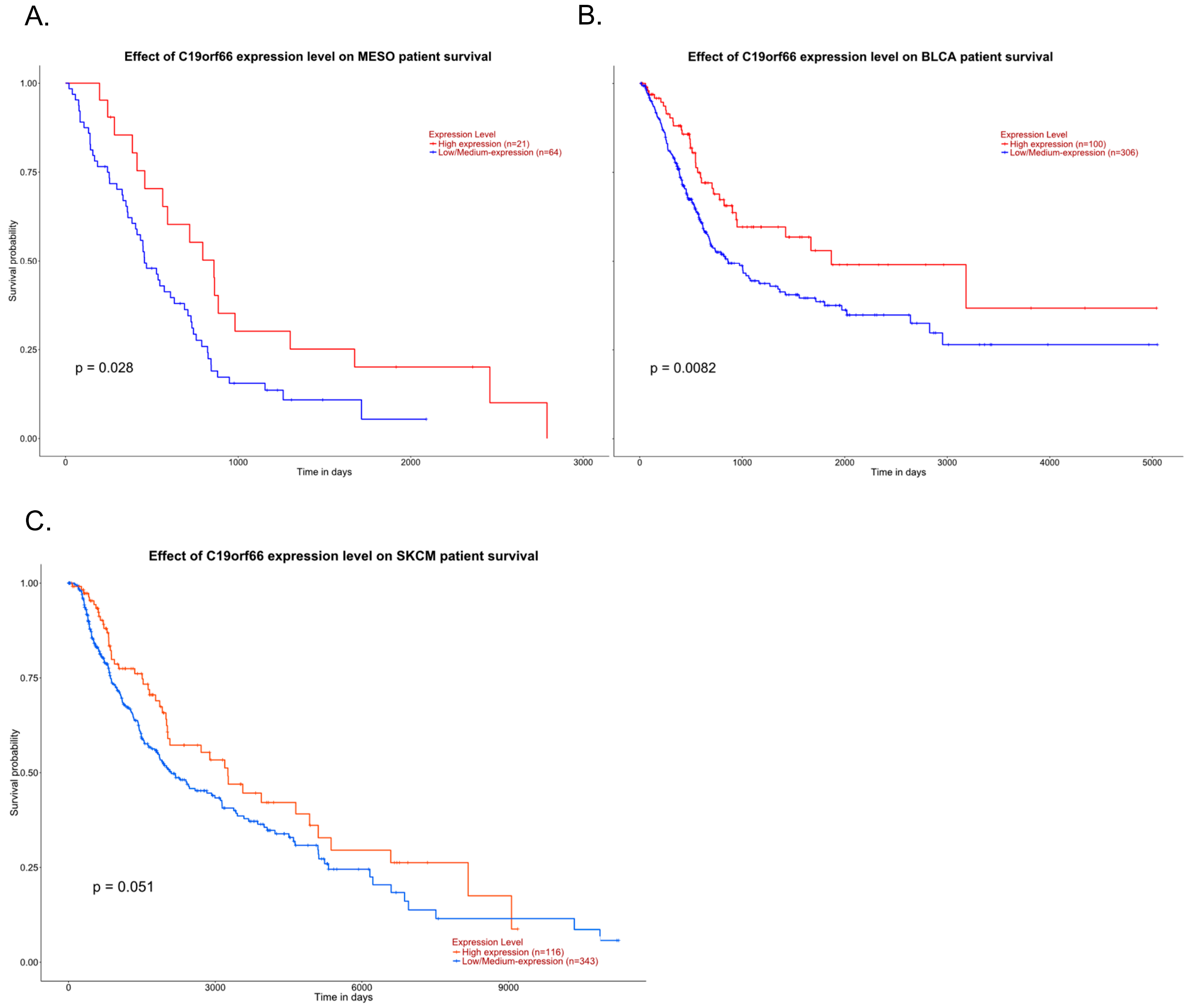

Supplemental Figure S7. Lower SFL expression decreases cancer patient survival.

Kaplan-Meyer survival plots comparing low and high-level Shiftless expression in (A) Lung mesothelioma, (B) Bladder urothelial carcinoma, and (C) Cutaneous melanoma.

	Normal			Cancer			Expression Change	
	Mean TPM	SD	N	Mean TPM	SD	N	Fold mean	% Change
Bladder (BLCA)	19.348	3.791429	9	12.09574	7.454498	432	0.625167	-37.4833
Breast	18.40608	6.584216	181	7.722593	5.202915	1253	0.419568	-58.0432
Colon	23.36609	9.238391	141	5.811777	4.246281	543	0.248727	-75.1273
Kidney (RCC)	20.41811	15.46794	28	8.765442	5.709652	615	0.429297	-57.0703
Liver	68.51388	24.48586	110	19.12129	11.57723	422	0.279086	-72.0914
Lung (MESO)	28.0186	10.74209	295	14.38403	6.311042	87	0.513374	-48.6626
Lung (LUAD)	28.0186	10.74209	295	8.378763	4.990853	598	0.299043	-70.0957
Prostate	30.51811	10.45481	100	6.631655	3.429536	556	0.217302	-78.2698
Skin (SKCM)	9.825529	3.824696	325	12.23792	7.540857	470	1.245522	24.55225
Average all							0.475232	-52.4768

Supplementary Table S1. Shiftless expression in common cancers.

Average transcripts per million reads (TPM) of Shiftless in Normal or cancerous tissues. Data mined from Genome Browser [1].

1. Lee, B.T.; Barber, G.P.; Benet-Pagès, A.; Casper, J.; Clawson, H.; Dlekhans, M.; Fischer, C.; Gonzalez, J.N.; Hinrichs, A.S.; Lee, C.M.; et al. The UCSC Genome Browser Database: 2022 Update. *Nucleic Acids Res.* **2022**, *50*, doi:10.1093/nar/gkab959.
